# Supplementary material for: Blind individuals’ enhanced ability to sense their own heartbeat is related to the thickness of their occipital cortex
Source: Cereb Cortex. 2024 Aug 16;34(8):bhae324. doi: 10.1093/cercor/bhae324 (PMC11329624; doi:10.1093/cercor/bhae324)
Supplement: SUPPLEMENTARY_MATERIAL_bhae324 [file supplementary_material_bhae324.docx]

**Supplementary material**

**
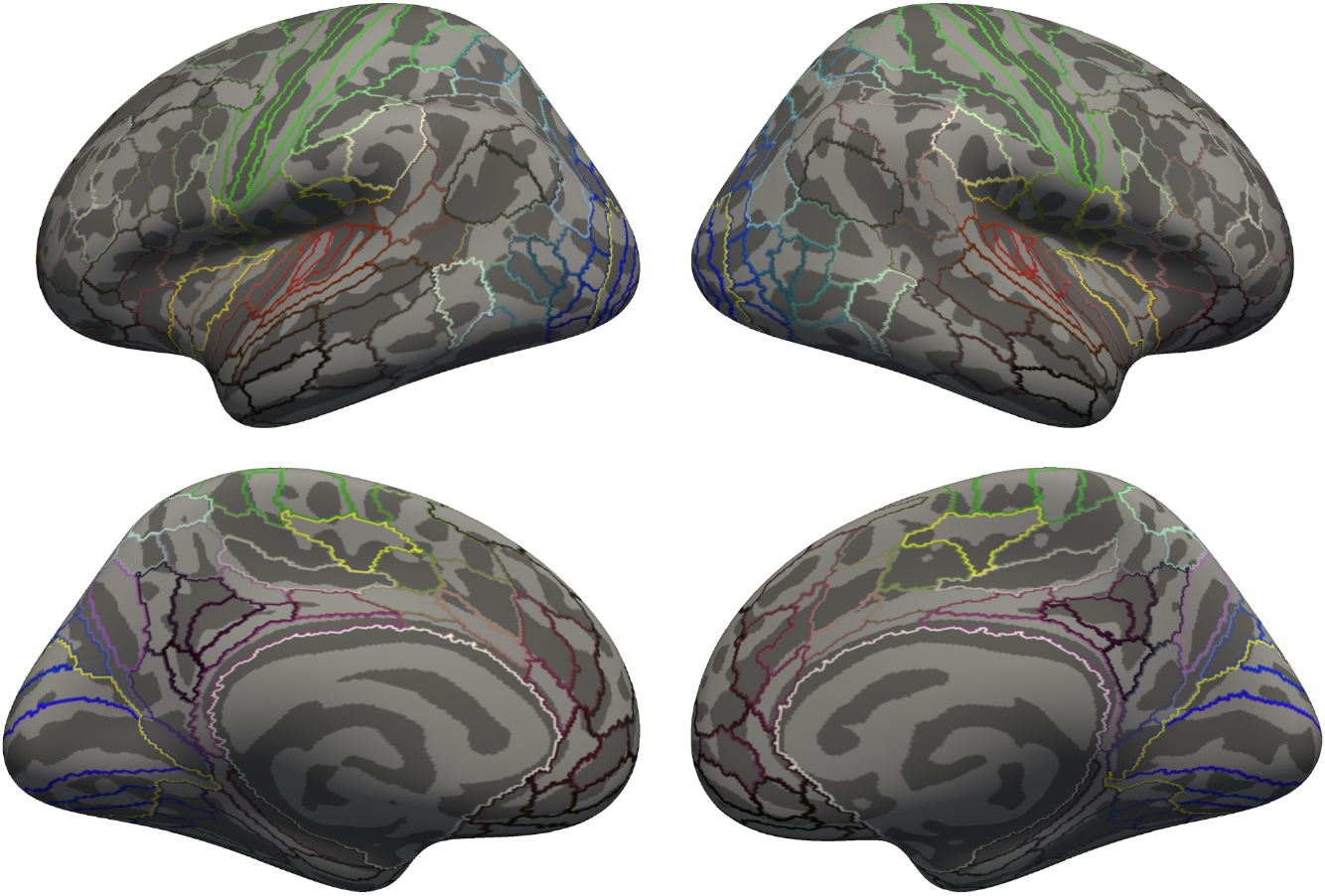
**

**Figure S1.** Inflated surface (dark gray: sulci, light gray: gyri) of the FreeSurfer standard brain. Colored lines indicate parcellations of the HCP-MMP1.0 atlas (Glasser et al. 2016); the yellow line demarcates the visual cortex and regions that have been shown to be involved in cardiac interoception (Schulz et al., 2016).


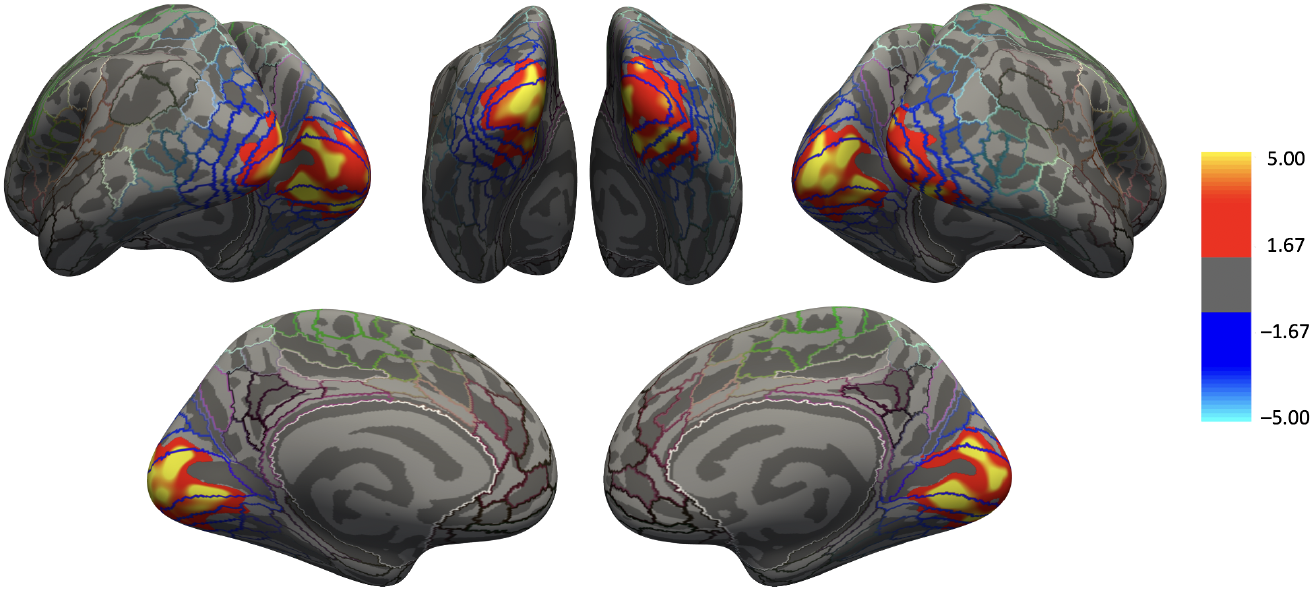


**Figure S2.** Group differences in visual cortical thickness with interoceptive accuracy as a covariate. Thresholded statistical significance maps (vertex-wise p < .01, cluster-wise p < .05, two-sided) display cortical thickness differences between congenitally blind individuals (CB group, n = 23) and sighted controls (SC group, n = 23). Maps are superimposed on the inflated surface (dark gray: sulci, light gray: gyri) of the FreeSurfer standard brain. Colored lines indicate parcellations of the HCP-MMP1.0 atlas (Glasser et al. 2016). Clusters with higher cortical thickness in the CB group are marked in red.


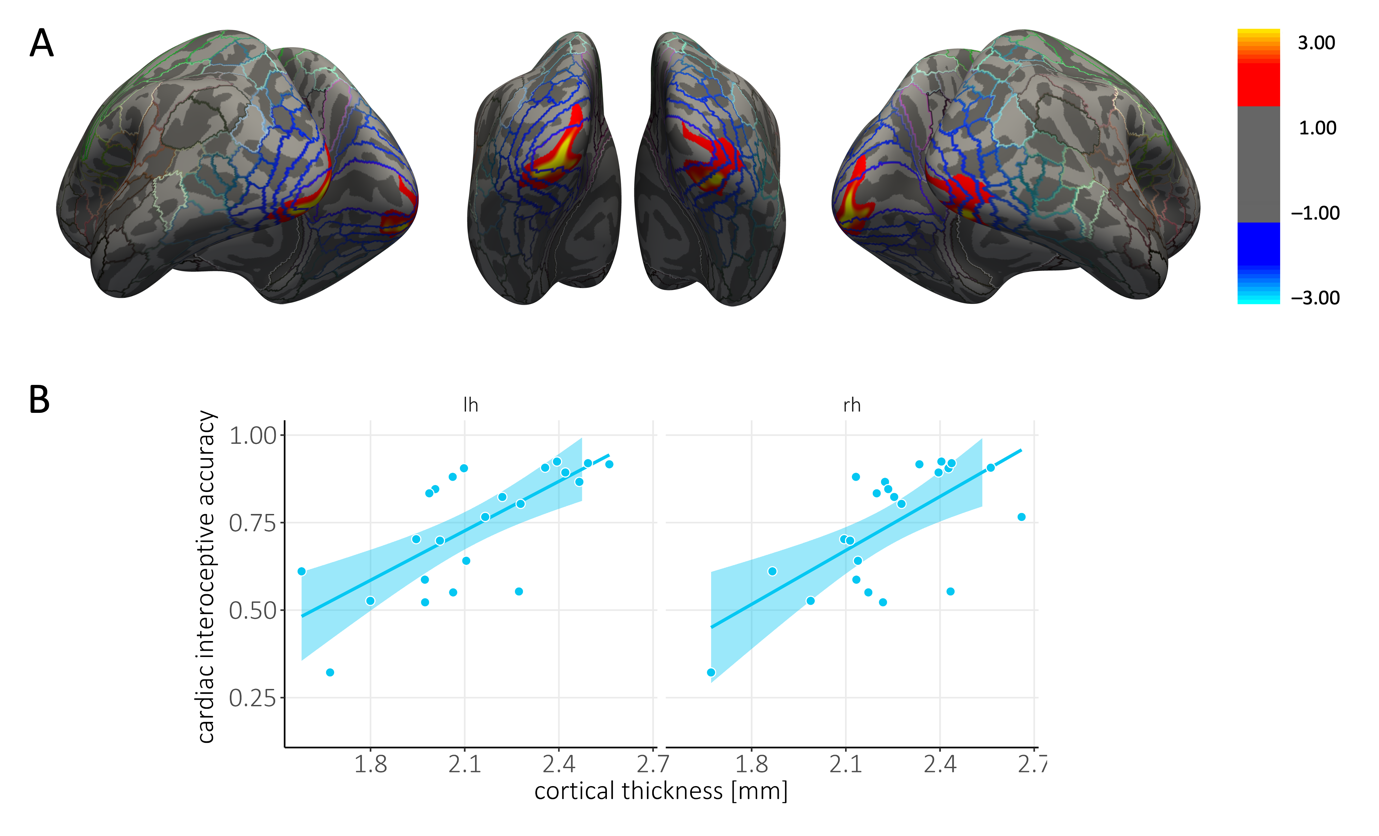


**Figure S3.** Positive correlation between visual cortical thickness and interoceptive accuracy in congenitally blind individuals. **A** Thresholded maps of correlation coefficients for cortical thickness with cardiac interoceptive accuracy in the group of congenitally blind individuals (CB group, n = 23). Maps are superimposed on the inflated surface (dark gray: sulci, light gray: gyri) of the FreeSurfer standard brain. Colored lines indicate parcellations of the HCP-MMP1.0 atlas (Glasser et al. 2016). The yellow outline demarcates our region of interest, including the visual cortex and regions of the brain that have been shown to be associated with cardiac interoception. Clusters that show a positive correlation between cortical thickness and interoceptive accuracy are shown in red/yellow. **B** Average cortical thickness extracted from the significant clusters plotted against the accuracy score from the cardiac interoception task. Note that we present interoceptive accuracy scores calculated using Schandry’s formula to allow comparison with previous studies. For the purposes of statistical analyses, these scores were standardized. Each dot represents one participant. The line represents the line of best fit.
